# TRACING THE COORDINATED CONTROL OF NAD+ HOMEOSTASIS USING C. ELEGANS

**DOI:** 10.1093/geroni/igad104.0595

**Published:** 2023-12-21

**Authors:** Melanie McReynolds

**Affiliations:** Penn State University, University Park, Pennsylvania, United States

## Abstract

Nicotinamide adenine dinucleotide (NAD+) is a vital coenzyme involved in redox reactions and cellular signaling. Its levels decline with age in humans, mice, and worms, leading to metabolic disturbances associated with age-related diseases. Interestingly, our studies in aged mice indicate that the rate of NAD+ synthesis remains unchanged, ruling out a lack of precursors as the cause of NAD+ decline. Instead, we found that tissues with lower NAD+ concentrations in aged mice exhibit higher fractional turnover, suggesting increased consumption as the primary driver of NAD+ decline during aging. This led us to hypothesize that the loss of NAD+ metabolic robustness and resiliency with age is linked to the hyper-activation and competition of NAD+-consuming enzymes. To investigate the physiological impact of NAD+ consuming enzymes on metabolic robustness, we used genetic tools and mass spectrometry in C. elegans. Surprisingly, we identified novel reproductive and aging phenotypes in mutant animals lacking specific NAD+ consuming enzymes, including Poly ADP-Ribose Polymerase (parp-1 and parp-2), TANKyrase (tank-1), and Toll and Interleukin 1 Receptor (tir-1). These mutants displayed significant reductions in fecundity and extensions in longevity. Additionally, employing High-Performance Liquid Chromatography coupled with Mass Spectrometry (HP-LC-MS), we examined the NAD+ metabolome across different developmental stages in our mutant strains, revealing notable metabolic disruptions associated with the loss of NAD+ consumers. In summary, our findings establish a novel connection between the hyper-activation of NAD+ degradation and the onset of reproductive aging. This research provides valuable insights into the role of NAD+ metabolism and its impact on aging-related processes.

